# Study on the Relationship between the Structure and Pyrolysis Characteristics of Lignin Isolated from Eucalyptus, Pine, and Rice Straw through the Use of Deep Eutectic Solvent

**DOI:** 10.3390/molecules29010219

**Published:** 2023-12-30

**Authors:** Tengfei Li, Xin Jin, Xinyao Shen, Hangdan Liu, Ruiping Tong, Xuzhen Qiu, Junfei Xu

**Affiliations:** Key Laboratory of Air-driven Equipment of Zhejiang Province, College of Mechanical Engineering, Quzhou University, Quzhou 324000, China; leetengfly@126.com (T.L.); jinyizhimianyang@126.com (X.J.); zjhzsxy4228@126.com (X.S.); lhd9442412370@126.com (H.L.); qqqiuxuzhen0614@163.com (X.Q.)

**Keywords:** lignin, deep eutectic solvent, pyrolysis, molecular structure, phenolic compounds

## Abstract

Understanding the pyrolysis product distributions of deep eutectic solvent (DES)-isolated lignins (DESLs) from different types of biomass is of great significance for lignin valorization. The structure and pyrolysis properties of DESLs obtained from eucalyptus (E-DESL), pine (P-DESL), and rice straw (R-DESL) were studied through the use of various methods such as elemental analysis, GPC, HS-GC, and NMR techniques, and the pyrolysis characteristics and product distributions of the DESLs were also further investigated through the use of TGA, Py-GC/MS, and tubular furnace pyrolysis. DESLs with high purity (88.5–92.7%) can be efficiently separated from biomass while cellulose is retained. E-DESL has a relatively low molecular weight, and P-DESL has a relatively higher hydrogen–carbon effective ratio and a lower number of condensation structures. The Py-GC/MS results show that, during DESL pyrolysis, the monomeric aromatic hydrocarbons, *p*-hydroxyphenyl-type phenols, and catechol-type phenols are gradually released when the guaiacyl-type phenols and syringyl-type phenols decrease with the rising temperature. 4-methylguaiacol and 4-methylcatechol, derived from the guaiacyl-type structural units, are positively correlated with temperature, which causes a significant increase in products with a side-chain carbon number of 1 from P-DESL pyrolysis. 4-vinylphenol, as a representative product of the R-DESL, derived from *p*-hydroxyphenyl-type structural units, also gradually increased. In addition, the P-DESL produces more bio-oil during pyrolysis, while gases have the highest distribution in E-DESL pyrolysis. It is of great significance to study the characteristic product distribution of lignin isolated through the use of DES for lignin directional conversion into specific high-value aromatic compounds.

## 1. Introduction

With the increasingly severe problems of resource consumption and environmental pollution, there is an urgent need to seek renewable energy sources to relieve the pressures on resources and the environment. Lignocellulosic biomass, which is renewable, environmentally friendly, abundant in nature, and widely distributed, is regarded as an ideal substitute for traditional fossil energy [1]. Lignin with aromatic properties is one of the most abundant natural renewable macromolecular compounds in nature and is a major component of lignocellulosic biomass. Lignin is usually produced as a by-product in the pulp and paper industry and in the biomass-refining process [2]. In particular, in the process of biomass component separation and hemicellulose component transformation, lignin with a high potential additional utilization value is produced as a by-product in large quantities. If it is left idle or burned, it will not only pollute the environment but also represent a great waste of high-quality renewable resources. Therefore, the processing and utilization of lignin as a by-product is very important for the efficient utilization of renewable resources and the relief of environmental pressures. According to the characteristics of lignin’s structure, it is important to degrade and convert lignin into aryl fuel and other high-added-value products via effective means, so as to partially replace petroleum and other fossil energy sources in the future to relieve energy pressures [3].

One of the main ways to achieve the high-value utilization of lignin is to degrade the lignin macromolecules formed from the phenylpropane basic structural units through the breakage of ether bonds or carbon–carbon bonds into low-molecular aromatic compounds via depolymerization strategies, such as oxidative degradation [4], hydrogen supply degradation [5], bio-enzymatic degradation [6], thermochemical degradation [7], and other depolymerization methods that can selectively break lignin’s intramolecular bonds. Due to the high bond dissociation energy required to break the bonds in lignin macromolecules [8], thermochemical depolymerization with good economy and high conversion efficiency is considered an effective method for lignin degradation [9]. In addition to the external conditions affecting the pyrolysis efficiency of lignin, such as the pyrolysis time, pyrolysis temperature, pyrolysis atmosphere, and the addition of catalysts, the molecular structure of lignin itself is also a key factor that affects the pyrolysis efficiency of lignin. The homolysis reaction of the representative β-O-4 bond in lignin and the side-chain fracture reaction of lignin in the pyrolysis process causes the rapid depolymerization of lignin macromolecules into low-molecular compounds [10]. In addition, the primary depolymerized low-molecular compounds are converted into aromatic monomers and oligomers due to the free-radical transfer reaction of the active oxygen-containing functional groups and the rearrangement of the functional groups in lignin during pyrolysis [11,12]. Furthermore, the interaction of oxygen-containing functional groups such as phenolic hydroxyl, aliphatic hydroxyl, and methoxyl during pyrolysis also has a significant influence on lignin pyrolysis efficiency and the formation of pyrolysis products [13,14,15]. Molecular weight distribution has also been proven to be an important factor affecting the formation of lignin pyrolysis products. For lignin, compared with a high molecular weight distribution, a low molecular weight distribution is more conducive to promoting the removal of methoxy during lignin pyrolysis, which results in an increase in low-oxygen pyrolysis compounds such as aromatic hydrocarbons and alkyl phenols [16,17,18]. Elemental composition is also considered to be an important factor affecting the conversion of lignin to energy. Having a high carbon–oxygen ratio (C/O) implies that lignin has a high heating value and can be used as an efficient fuel [19], and the hydrogen–carbon effective ratio (H/C_eff_) has been proven to contribute to the formation of lignin pyrolysis bio-oils and aromatic products [20].

The economical and effective separation of lignin from biomass feedstocks is an important prerequisite for the stable and efficient conversion of lignin into aromatic compounds [21]. Many lignin separation techniques have been developed over the course of long-term research. The large amount of alkali lignin produced in the pulping process is the main raw material of lignin conversion engineering, but its structure is damaged significantly in the harsh high-temperature alkali treatment process, which limits its efficient conversion in the pyrolysis process [22]. A method of obtaining Klason lignin is to completely degrade carbohydrates through acid hydrolysis to achieve the separation of lignin. However, the separation process is accompanied by the formation of a large number of condensation structures and the loss of all carbohydrates, which is contrary to the economic utilization of lignin. Enzymatic/mild acid hydrolysis lignin (EMAL) can be separated from biomass feedstocks with little damage to the original structure of lignin via an enzymatic method combined with mild acid hydrolysis. Unfortunately, the high cost of enzymes and the complete loss of cellulose during processing limit the development of EMAL to the exploration of the lignin pyrolysis conversion principle in the laboratory [23]. Traditional ionic liquids have also proven to be efficient solvents for lignin separation. However, the poor biocompatibility and economy of ionic liquids have affected their large-scale use in the separation of biomass components [24]. As a substitute for traditional ionic liquids, DES combines sustainability, biocompatibility, degradability, and economy and is efficient in the separation of lignin from biomass feedstocks under relatively mild conditions (<120 °C) [25,26]. Compared with alkali lignin and other industrial lignin, the lignin isolated from biomass feedstocks via DES treatment has a relatively low condensation degree compared with that from industrial lignin such as kraft lignin, and the DES can also be reused after the treatment of biomass [27,28]. In comparison to the more substantial research on the pyrolysis of the typical lignin representatives, such as alkali lignin and EMAL, the investigation of the pyrolysis characteristics of lignin macromolecules with high-quality molecular properties isolated from different representative biomass feedstocks via DES still needs to be further developed.

To investigate the relationship between the structure and pyrolysis characteristics of lignin isolated via DES treatment, three lignin samples were isolated from eucalyptus, pine, and rice straw through the use of DES treatment. The molecular compositions of three lignin samples isolated via DES (DESLs) were evaluated via elemental analysis and gel permeation chromatography (GPC). In addition, the main bonds and functional groups were identified via ^31^P-nuclear magnetic resonance (NMR), headspace gas chromatography (HS-GC), and 2D heteronuclear single quantum correlation (HSQC) NMR techniques. Furthermore, the thermochemical degradation characteristics of the DESLs were also determined via thermogravimetric analysis (TGA), pyrolysis (Py)-GC/MS, and tubular furnace pyrolysis.

## 2. Results and Discussion

### 2.1. Effect of Deep Eutectic Solvent on the Isolation of Lignin from Biomass Feedstocks

Three representative biomass feedstocks, including eucalyptus, pine, and rice straw, contain considerable amounts of lignin (20.7–26.8%), as shown in Table 1. This suggests that the amount of lignin in these biomass feedstocks, which exist in large quantities around the world, is tremendous after being isolated in the biomass-processing industry, and this has not been ignored in the replacement of non-renewable aromatic chemical raw materials.

DES composed of choline chloride and lactic acid showed good dissolution of lignin in the biomass feedstocks, which was confirmed by the result that more than 80% of lignin in all three biomass feedstocks was isolated after being treated with DES at 120 °C for 6 h (Table 2). After DES treatment, the deep degradation of most hemicellulose in biomass feedstocks and the breakage of lignin–carbohydrate complex links resulted in the release of a large amount of high-purity (88.5–92.7%) lignin [29], as shown in Table 3. However, the damage to the cellulose portion via DES treatment was not significant (Table 4). These results indicated that the separation of the three main components of the biomass feedstocks was able to be completed with DES treatment under mild conditions in a relatively short time, and high-purity lignin was obtained at a high yield while most cellulose components were retained, which was very favorable for the subsequent high-value utilization of each component of the biomass feedstocks. These results suggest that DES had obvious advantages in terms of reaction conditions, separation efficiency, and lignin purity in comparison to industrial lignin and enzymatic/mild acid hydrolysis lignin (EMAL) [23].

### 2.2. The Structural Characterization of Lignin Isolated through the Use of Deep Eutectic Solvent

To further investigate the structural properties of lignin isolated from eucalyptus, pine, and rice straw via DES treatment, various structural analysis methods were used to determine the elemental composition, molecular weight distribution, main oxygen-containing functional groups, and intermolecular bonds of E-DESL, P-DESL, and R-DESL.

#### 2.2.1. Elemental Analysis

The elemental compositions of the E-DESL, P-DESL, and R-DESL are listed in Table 5. The high carbon content (61–63%), low nitrogen content (0.1–0.6%), and almost negligible sulfur suggest that DESLs are a pollution-free renewable fuel with high calorific value. Compared with the E-DESL and R-DESL, the content of carbon in the P-DESL was relatively low and the content of hydrogen was relatively high, which caused the higher hydrogen-to-carbon effective ratio (H/C_eff_) of the P-DESL. The H/C_eff_ is a significant indicator related to the formation of aromatic products and the generation of pyrolysis char in lignin pyrolysis. The higher heating value (HHV) of the DESLs was between 24.43 to 24.73 MJ/Kg, which suggested that the DESLs are a potentially highly valuable raw material for conversion into high-calorific-value fuels.

#### 2.2.2. 2D HSQC NMR Analysis

**Figure 1:**
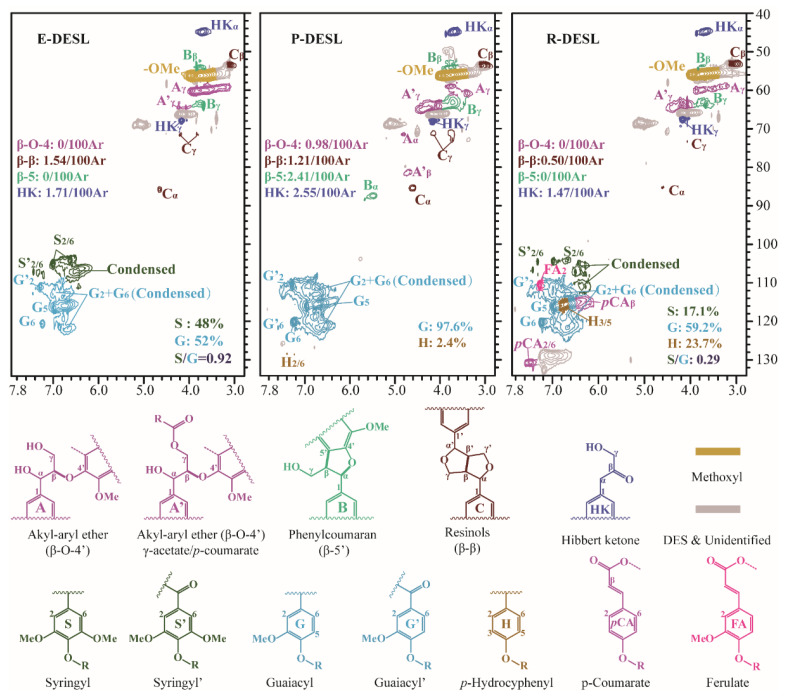
The 2D ^13^C-^1^H (HSQC) spectra of the DESLs, and the chemical structure corresponding to each spectral peak.

Next, 2D HSQC NMR was applied for the determination of the DESLs’ structure. The results of the retrieval, determination, and calculation of the main structures of the E-DESL, P-DESL, and R-DESL are shown in Figure 1, which are based on the results of previous studies [30]. As shown in Figure 1, the E-DESL was composed of a guaiacyl (G)-type unit and a syringyl (S)-type unit; the G-type basic structural unit played a dominant role in the composition of the P-DESL. However, a large number of *p*-hydroxyphenyl (H)-type units were present in the composition of the R-DESL. This indicated that the basic benzene ring skeleton of the DESLs was not significantly damaged by DES treatment.

As shown in Figure 1, the β-O-4 bond was completely absent in the E-DESL and R-DESL, and only 0.98/100Ar of the β-O-4 bond was retained in the P-DESL, which indicated that the β-O-4 bond was particularly unstable in the DESLs during DES treatment. The β-5 bonds in the E-DESL and R-DESL were also completely destroyed during DES treatment, but about 2.41/100Ar of the β-5 bond was still present in the P-DESL, which suggested that the β-5 bond was more stable in the P-DESL than in the E-DESL and R-DESL. In addition, a small number of β-β bonds in all three DESLs were retained. This indicated that DES treatment was unable to completely destroy the β-β bond in the DESLs. It was intriguing to find that the Hibbert ketone (HK) structure was present in three DESLs, which was significantly different from the lignin extracted via other methods [31,32]. This might be due to the breakage and oxidation of the β-O-4 bond in the DESLs during DES treatment [27].

#### 2.2.3. The Quantitative Analysis of the Main Functional Groups of Lignin Isolated through the Use of Deep Eutectic Solvent

As shown in Table 6, the total phenolic hydroxyl of the three DESLs was between 5.24 to 5.58 mmol/g, and the total hydroxyl content was between 7.05 to 7.51 mmol/g, which was higher than the hydroxyl content in the lignin isolated via the other methods, such as sulfate pulping black liquor (kraft lignin), but the content of condensed phenolic hydroxyl in the DESLs was less than that in kraft lignin.

It can be seen in Table 6 that considerable amounts of guaiacyl phenolic hydroxyl (G) and syringyl phenolic hydroxyl (S) and a small amount of *p*-hydroxyphenyl phenolic hydroxyl (H) in the E-DESL were determined, and the G/S/H was 4.9/5.5/1. The G/S/H of the R-DESL was determined to be 2.0/0.6/1. However, the phenolic hydroxyl of the P-DESL was composed of G and negligible amounts of H. This was consistent with the 2D HSQC NMR analysis results. The methoxyl group of the DESLs was also detected, and the order of methoxyl content of three types of DESLs was found as follows: E-DESL > P-DESL > R-DESL. This was mainly because compared with the P-DESL, the S-type structural unit containing two methoxy groups in the E-DESL was dominant, while the H-type structural unit without a methoxy group in the R-DESL was an important part.

#### 2.2.4. Analysis of the Molecular Weight of Lignin Isolated through the Use of Deep Eutectic Solvent

The molecular weight of the three DESLs was also determined via GPC, and the results are shown in Table 7. It was found that the Mn of the E-DESL and R-DESL was lower than that of the P-DESL, which suggested that the E-DESL and R-DESL macromolecules were more prone to be depolymerized to lower molecular weight fragments during DES treatment compared with the P-DESL. This might be because the bonds between the basic structural units of the P-DESL were more difficult to break during DES treatment than that of the E-DESL and R-DESL based on the analysis results of 2D HSQC NMR. In addition, the Mw of the E-DESL isolated from hardwood was significantly greater than that isolated from hardwood via enzymatic/mild acid hydrolysis [33] and was similar to that isolated from hardwood through the use of the organic solvent method [34]. It is worth mentioning that the Mw of the R-DESL was relatively large compared with that of the E-DESL, which resulted in a relatively higher polydispersity index (PDI) for the R-DESL. Furthermore, the Mw of the P-DESL was significantly higher than that of the E-DESL and R-DESL, and the PDI of the P-DESL was 2.43. These results indicated that compared with the R-DESL and P-DESL, the E-DESL molecular weight was more uniform.

### 2.3. The Thermochemical Depolymerization Analysis of Lignin Isolated through the Use of Deep Eutectic Solvent

**Figure 2:**
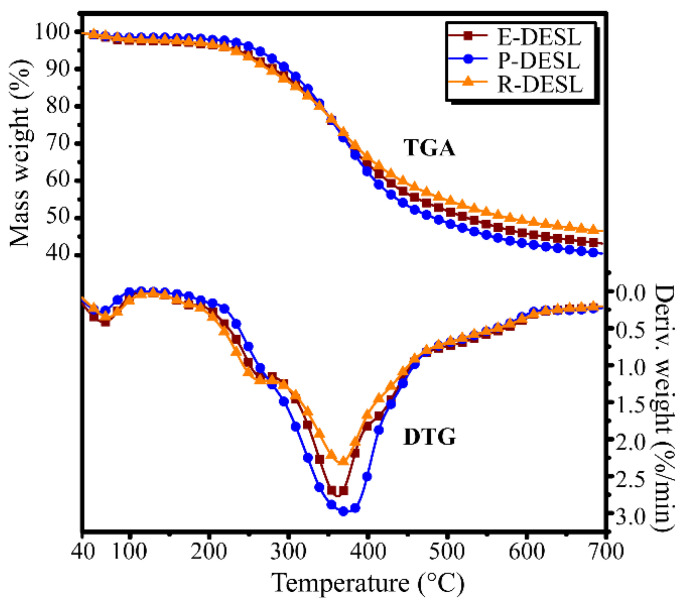
Curves of TGA and DTG for the different DESLs.

Thermogravimetric analysis (TGA) was applied for the determination of the thermal degradation properties of the DESLs, and the TGA and derivative thermogravimetic analysis (DTG) curves of the DESLs were obtained. As shown in Figure 2, negligible moisture volatilized between 40 and 120 °C according to the appearance of a small degradation peak at this temperature range. The peaks between 200 and 300 °C appeared in the E-DESL and R-DESL curves, but not in the P-DESL DTG curves, which indicated that the residual carbohydrates remained in small amounts in the E-DESL and R-DESL but were completely removed in the P-DESL [35,36].

The main pyrolysis of the DESLs occurred at temperatures between 300 to 600 °C, at which the DESLs were degraded into low-molecular compounds and were released in gaseous form due to the bonds breaking and functional group reforming reactions. It was interesting to find that compared with the E-DESL and R-DESL, the maximum degradation rate (MDR) of the P-DESL was the highest, and the temperature at which the MDR (T_max_) of the P-DESL occurred was higher than that of the E-DESL and R-DESL. This suggested that compared with the E-DESL and R-DESL, the P-DESL had higher thermal stability, which resulted in the P-DESL producing less pyrolysis coke. Compared with the E-DESL, the R-DESL had a higher MDR and a lower T_max_. This suggested that the R-DESL with a more complex composition of basic structural units released less volatile matter during pyrolysis.

**Figure 3:**
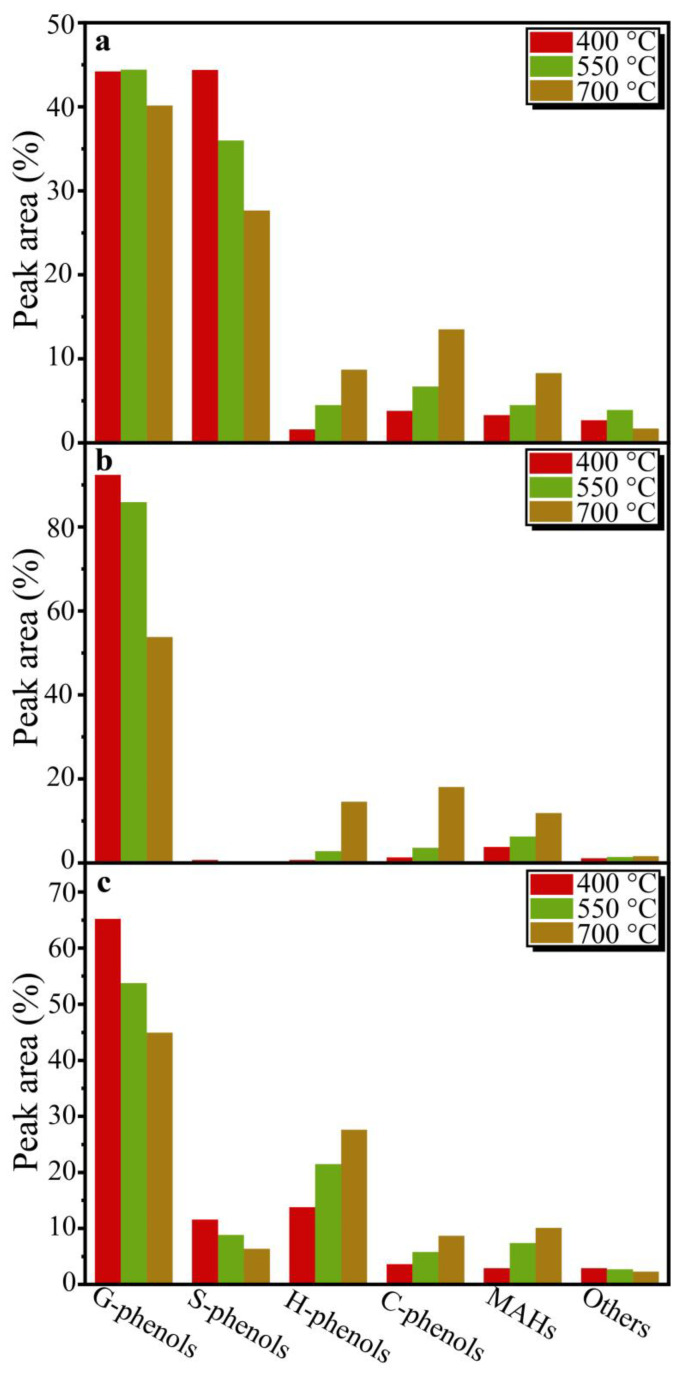
Product distributions from the E-DESL, P-DESL, and R-DESL pyrolysis at different temperatures. (**a**): E-DESL pyrolysis products; (**b**): P-DESL pyrolysis products; (**c**): R-DESL pyrolysis products. G-phenols: guaiacyl-type phenols; S-phenols: syringyl-type phenols; H-phenols: *p*-hydroxyphenyl-type phenols; C-phenols: catechol-type phenols; MAHs: monomeric aromatic hydrocarbons.

**Figure 4:**
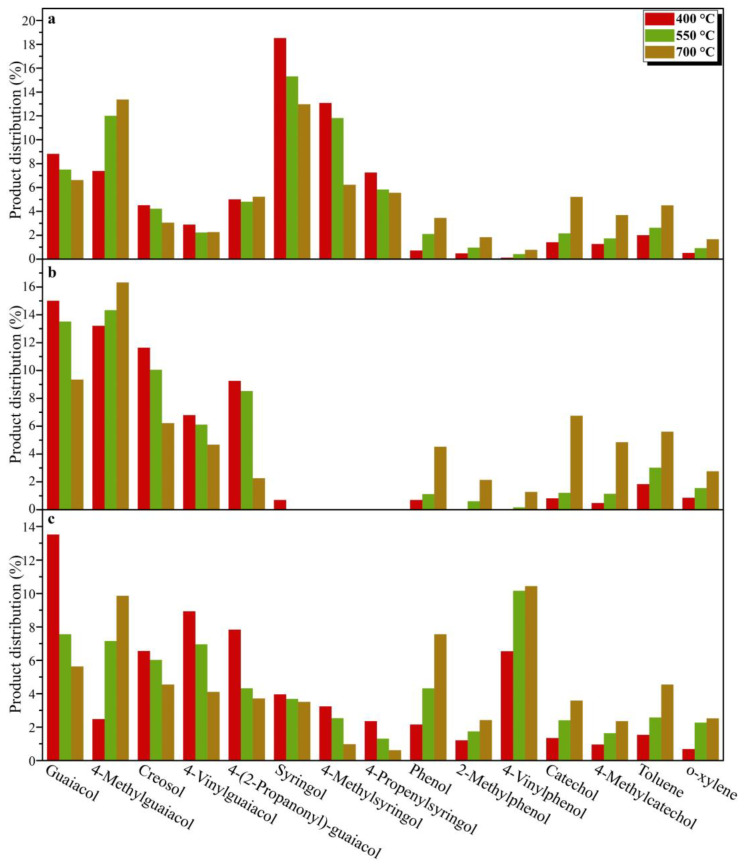
The distributions of the main products from E-DESL, P-DESL, and R-DESL pyrolysis at different temperatures. (**a**) E-DESL pyrolysis products; (**b**) P-DESL pyrolysis products; (**c**) R-DESL pyrolysis products.

**Figure 5:**
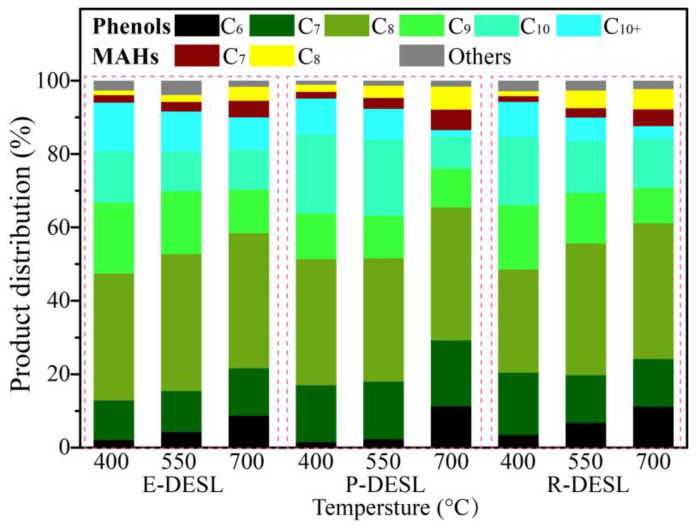
The carbon distribution of products from E-DESL, P-DESL and R-DESL pyrolysis under different temperatures.

**Figure 6:**
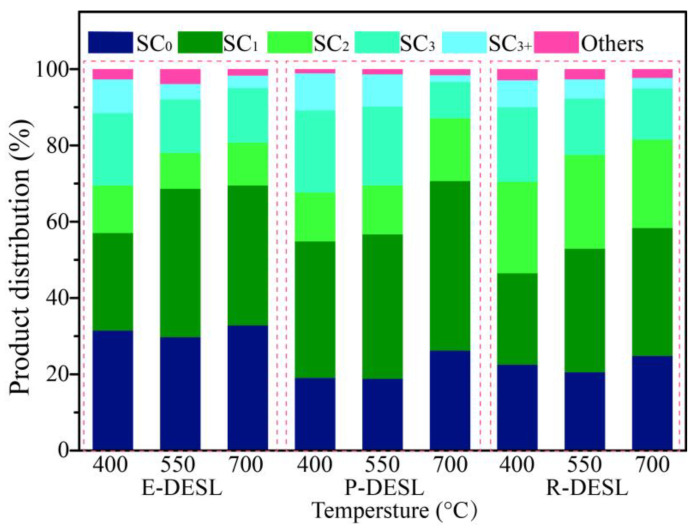
The carbon number distributions of the longest side-chain of products from E-DESL, P-DESL, and R-DESL pyrolysis under different temperatures.

To further investigate the relationship between the structure of lignin isolated from different types of biomass feedstocks via DES and the distributions of pyrolysis products, fast pyrolysis experiments for the DESLs at 400 °C, 550 °C, and 700 °C were carried out, and about 42 of the products were retrieved and sorted via GC/MS, as shown in the Supplementary Materials. Since the products from lignin pyrolysis were mainly composed of aromatic compounds and the types of pyrolysis products were closely related to the lignin structural units, the pyrolysis products of the DESLs were categorized into guaiacyl type (G)-phenols, *p*-hydroxyphenyl type (H)-phenols, syringyl type (S)-phenols, catechol type (C)-phenols, monomeric aromatic hydrocarbons (MAHs), and others (Figure 3). In addition, the distribution of the main DESL pyrolysis products at different temperatures is shown in Figure 4. The carbon number distribution of pyrolysis products derived from the three DESLs is shown in Figure 5. The carbon number distribution of the longest side-chain of products from DESL pyrolysis under different temperatures is also shown in Figure 6.

After the identification of products from DESL pyrolysis at 400 °C, the G-phenols and S-phenols were the main components of E-DESL pyrolysis products (Figure 3a), G-phenols were the dominant component of P-DESL pyrolysis products (Figure 3b), and in addition to G-phenols, H-phenols also played a significant role in the R-DESL pyrolysis products, as shown in Figure 3c, which is mainly due to the thermal fracture of the linkages between the basic structural units of DESLs with poor thermal stability at 400 °C based on the TGA results. It is worth noting that the 4-(2-propanonyl)-guaiacol as the main pyrolysis product of DESLs was produced in large quantities at the pyrolysis temperature of 400 °C, and the distribution of 4-(2-propanonyl)-guaiacol in the three DESLs’ pyrolysis products was consistent with the HK content shown in the 2D HSQC NMR results.

With the pyrolysis temperature gradually increased, more products are released during lignin pyrolysis because of free radical recombination and side-chain breakage [37]. As shown in Figure 3a and Figure 4a–c, compared with G-phenols, the content of S-phenols including syringol, 4-methylsyringol, 4-acetylsyringol, and 4-propenylsyringol was greatly affected by the pyrolysis temperature. This indicated that the S-type structural unit in the E-DESL was thermally unstable and easily degraded by heating during the pyrolysis process. The H-phenols, such as phenol, 2-methylphenol, and 4-vinylphenol, and the C-phenols, including 4-methylcatechol and catechol, were gradually released due to the drastic demethoxylation and demethylation as the pyrolysis temperature increased [38]. The yield of MAHs, such as *o*-xylene and toluene, was also significantly increased due to the occurrence of a deep deoxidation reaction under the pyrolysis condition of 700 °C [39]. As shown in Figure 3b,c, compared to the E-DESL, the yield of G-phenols from P-DESL and R-DESL pyrolysis was obviously affected by the pyrolysis temperature. However, the formation of H-phenols, C-phenols, and MAHs from P-DESL and R-DESL pyrolysis was positively correlated with the pyrolysis temperature. The formation of S-phenols from R-DESL pyrolysis was similar to that from E-DESL pyrolysis.

As shown in Figure 5, the pyrolysis products with a carbon number more than C_9_ were produced in large quantities at the pyrolysis temperature of 400 °C, and their content showed a gradually decreasing trend due to the degradation of G-phenols, S-phenols, and pyrolysis products containing long side-chains with the increase in pyrolysis temperature. At the same time, the pyrolysis products without -OCH_3_ and with short side-chains, such as H-phenols, C-phenols, and MAHs, gradually increased due to the demethoxylation reaction, and the side-chain fracture reaction intensified with the increase in pyrolysis temperature [40], resulting in the carbon number distribution of pyrolysis products gradually concentrating in the range of C_6_–C_8_. At the pyrolysis temperature of 700 °C, the content of pyrolysis products with C_6_ was significantly increased compared with the content of that from pyrolysis at 400 °C, which resulted from the massive release of phenol and catechol, as shown in Figure 4a–c and Figure 5. The effect of pyrolysis on the lignin side-chain was also further investigated, and it can be seen in Figure 4a and Figure 6 that due to the rapid release of syringol and guaiacol from E-DESL pyrolysis at 400 °C, the products with the carbon number distribution of the longest side-chain (SC) of 0 were significantly greater than those from P-DESL and R-DESL pyrolysis. Since the rapid release of phenol mainly occurred in the process of high-temperature pyrolysis, the content of pyrolysis products with SC_0_ reached a peak at 700 °C pyrolysis. It was intriguing to find that although the G-phenols and S-phenols gradually decreased with the increase in pyrolysis temperature, 4-methylguaiacol with SC_1_ belonging to the G-phenols showed a positive correlation with pyrolysis temperature. In addition, the content of catechol derived from the degradation of the G-type structural unit was also positively correlated with the pyrolysis temperature (Figure 4a–c). These results indicated that the C_α_–C_β_ of the G-type structural unit in the DESLs was easily broken via heating during pyrolysis, and therefore, the 4-methylguaiacol and 4-methylcatechol with strong thermal stability were formed. This was also the main reason as to why the pyrolysis products with SC_1_ increased with the pyrolysis temperature increasing. The trend of pyrolysis products with SC_1_ changing with the temperature was more significant in the distribution of products from the pyrolysis of the P-DESL consisting mainly of G-type structural units, as shown in Figure 6. Different from the E-DESL and P-DESL, the content of products with SC_2_ from E-DESL pyrolysis significantly increased. This result was mainly attributed to the release of a large amount of 4-vinylphenol directly derived from the H-type structural unit with SC_2_ during E-DESL pyrolysis.

**Figure 7:**
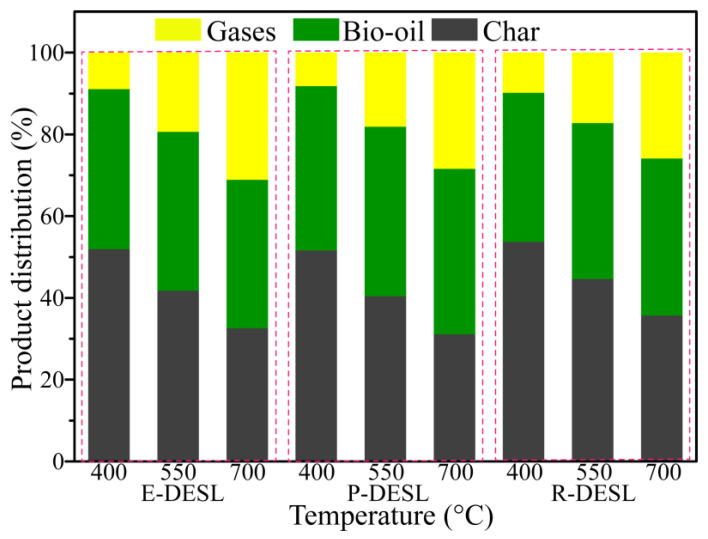
The yield of three-phase pyrolysis products from E-DESL, P-DESL, and R-DESL pyrolysis at different temperatures.

The distributions of three-phase products from DESL pyrolysis at different temperatures were also investigated. As shown in Figure 7, with the pyrolysis temperature increasing, the content of char gradually decreased, and the content of the gases had a gradually increasing trend due to the breakage of side-chains and oxygen-containing bonds between the basic structural units of the DESLs. This means that the yield of bio-oil derived from P-DESL pyrolysis was greater than that from the E-DESL and R-DESL. In addition, the yield of char derived from the P-DESL was lower than that from the other DESLs. This was probably attributed to several reasons: in comparison with the E-DESL and R-DESL, the P-DESL had lower content of condensed structures (Table 6); a small number of β-O-4 bonds still existed in the P-DESL; and the higher H/C_eff_ was another reason for the char decrease in P-DESL pyrolysis [41,42]. It can also be seen in Figure 7 that the content of pyrolysis gases released mainly through the degradation of the lignin side-chain and the removal of methoxy occupied the highest proportion in the E-DESL but the lowest proportion in the R-DESL when the pyrolysis temperature was above 550 °C. This might be due to the existence of a considerable content of S-type units to provide a large amount of methoxy for the E-DESL (Table 6).

## 3. Materials and Methods

### 3.1. Materials

The representative lignocellulosic biomass feedstocks, eucalyptus wood chips (3 cm × 1.5 cm) and pine wood chips (0.3–0.5 cm), were provided by the city of Zhanjiang, Guangdong province, and the rice straw powders (0.4–0.8 mm) were provided by the city of Quzhou, Zhejiang province. These three air-dried biomass feedstocks were cleaned and ground to particle sizes of 0.25-0.38 mm and then dried at 105 °C for 4 h for the lignin isolation experiments. The choline chloride was purchased from Shanghai Maclean Biochemical Technology Co., Ltd. (Shanghai, China), and the other chemicals, including diethyl ether, lactic acid, and ethanol, were supplied by Sinopharm Chemical Reagent Co., Ltd. (Guangzhou, China).

### 3.2. Preparation of the Lignin Isolated through the Use of Deep Eutectic Solvent

The deep eutectic solvent (DES) for lignin isolation was synthesized from lactic acid and choline chloride (molar ratio of 2:1) at 60 °C for 2 h. For each of the lignin isolation experiments, the prepared DES and the biomass feedstocks were mixed in a 1000 mL flask at a mass ratio of 20:1 and reacted at 120 °C for 6 h under magnetic stirring. After the reaction, the mixture of DES and the solid fractions was immediately vacuum filtered using a G_2_ filter to separate the DES soluble fractions and solid residues. The liquid fractions obtained via filtration were collected in a beaker, and then, a sufficient amount of deionized water was added to the beaker containing the DES to precipitate the lignin dissolved in the DES. The lignin precipitates collected via centrifugation were repeatedly washed at least three times with water/ethanol (9/1). The washed lignin was further rinsed with anhydrous diethyl ether to remove the soluble impurities and the obtained purified lignin was then freeze-dried for 48 h. The lignin samples isolated from eucalyptus, pine, and rice straw via DES treatment were named E-DESL, P-DESL, and R-DESL, respectively.

### 3.3. Structural Characterization of Lignin Isolated through the Use of Deep Eutectic Solvent

#### 3.3.1. Elemental Analysis

The constituent elements in the three DESLs were identified by a Vario EL elemental analyzer (Elementar, Hesse, Germany).

#### 3.3.2. 2D HSQC NMR Analysis

^1^H-^13^C 2D HSQC NMR was conducted on an ADVANCE III 600 MHz spectrometer (Bruker, Karlsruhe, Germany) with a 6 s relaxation delay for 10 h for the structural characterization of the three DESLs. The preparation of lignin samples for NMR detection and the specific parameter setting for NMR detection were carried out based on the previous references [33].

#### 3.3.3. ^31^P-NMR Analysis

The determination of the hydroxyl groups of the three DESLs was carried out via quantitative ^31^P-NMR on an ADVANCE III 600 MHz spectrometer. The phosphating process of the hydroxyl groups in the DESLs and the setting of the NMR test parameters were performed according to a previous study [43].

#### 3.3.4. HS-GC Analysis

The methoxy groups of the three DESLs were determined via headspace gas chromatography (HS-GC). The methoxy groups were determined as follows: 500 μL of 57% HI and 10 mg of DESLs were mixed thoroughly into a headspace test bottle, and the bottle was sealed. The sealed bottle reacted at 130 °C for 0.5 h and was then cooled to indoor temperature. Approximately 500 μL of 6 mol/L sodium hydroxide solution was added into the bottle. Subsequently, the bottle was placed into a headspace autosampler for detection [44].

#### 3.3.5. GPC Analysis

The molecular weight distributions of the three DESLs were determined via gel permeation chromatography (GPC) (Agilent, Palo Alto, CA, USA). Approximately 5 mg of each DESL was added to 5 mL of THF, which was filtered via a nylon filter (0.45 μm), and then injected into columns for the test with THF at 30 °C. Polystyrene standards (500 to 50,000 g/mol) were applied for the calibration of molecular weight.

### 3.4. Thermochemical Analysis of Lignin Isolated through the Use of Deep Eutectic Solvent

#### 3.4.1. TG Analysis

The three DESLs completely dehydrated by vacuum drying were tested via a TG Q50 analyzer (TA, Milford, CT, USA). Approximately 5 mg of each DESL was tested in the temperature range (40 °C to 700 °C) at 10 °C/min with a N_2_ (purity of 99.999%).

#### 3.4.2. Pyrolysis-GC/MS Analysis

A high-temperature Curie point CDS 5200 pyrolyzer (CDS Analytical, Oxford, MS, USA) was used for the investigation of volatiles from the DESLs’ fast pyrolysis. Approximately 0.4 mg of the DESLs was loaded on a pyrolysis crucible. The pyrolysis temperature was set from 25 °C to a different final temperature with a flash rate and a 30 s residence time. The evolved volatiles were determined via GC/MS (7890B/5977A) (Agilent, Palo Alto, CA, USA), with the pyrolysis conditions taken from a previous reference [23].

#### 3.4.3. Tubular Furnace Pyrolysis Analysis

The three-phase products, such as gases, solid char, and liquid bio-oil from the DESLs pyrolysis, were collected and quantitatively measured during the tubular furnace pyrolysis experiment. Before the DESL pyrolysis experiment, the air in the tube was replaced by highly purified nitrogen in advance to form a pure nitrogen atmosphere inside the quartz tube, and the heating area where the quartz tube was loaded was heated to a specified temperature and maintained for a period of time. Then, 500 mg of each DESL was placed in a quartz tube for pyrolysis. After the experiment, the three-phase pyrolysis products were collected separately and measured, and the relevant methods used referred to those in a previous reference [45].

## 4. Conclusions

To better understand the relationship between the structure of lignin isolated from different biomass feedstocks through the use of DES and the formation of pyrolysis products, three DESLs, including the E-DESL, P-DESL, and R-DESL, were isolated from eucalyptus, pine, and rice straw through the use of DES, respectively. The structure of the DESLs was studied via elemental analysis, GPC, HS-GC, and NMR techniques, and the pyrolysis characteristics and product distributions of the DESLs were also further investigated via TGA, Py-GC/MS, and tubular furnace pyrolysis. The results showed that DES was able to efficiently separate high-purity (>88%) lignin from the biomass while cellulose was retained. The E-DESL had a lower molecular weight, and the P-DESL had a higher H/C_eff_ and fewer condensation structures. The G-phenols and S-phenols were thermally unstable, and the release of the H-phenols, C-phenols, and MAHs increased with the rising temperature. The 4-methylguaiacol and 4-methylcatechol were positively correlated with temperature, which caused a significant increase in products with SC_1_ from P-DESL pyrolysis. The 4-vinylphenol, as a representative product of the R-DESL, also gradually increased. In addition, more bio-oil produced from P-DESL pyrolysis and gases had the highest distribution in E-DESL pyrolysis.

## Figures and Tables

**Table 1:** Composition analysis of biomass feedstocks.

Composition (%)	Eucalyptus	Pine	Rice Straw
Glucan	51.8 ± 0.8	53.2 ± 1.0	48.3 ± 0.7
Xylan	13.1 ± 0.2	2.9 ± 0.1	14.5 ± 0.2
Galactan	2.3 ± 0.1	1.2 ± 0.1	2.5 ± 0.1
Arabinan	0.6 ± 0.1	0.4 ± 0.1	1.4 ± 0.1
Mannnan	3.1 ± 0.1	12.4 ± 0.2	3.6 ± 0.1
Acid-soluble lignin	22.8 ± 0.3	24.5 ± 0.3	19.2 ± 0.2
Acid-insoluble lignin	2.1 ± 0.1	2.3 ± 0.1	1.5 ± 0.1
Total lignin	24.9 ± 0.4	26.8 ± 0.4	20.7 ± 0.3
Extraction	2.7 ± 0.1	2.4 ± 0.1	3.6 ± 0.1
Ashes	0.9 ± 0.1	0.6 ± 0.1	1.7 ± 0.1

**Table 2:** Separation yield of solid residue and lignin from biomass treated with deep eutectic solvents at 120 °C for 6 h.

Component	Eucalyptus	Pine	Rice Straw
Solid residue (g/100 g biomass material)	43.2 ± 0.6	46.1 ± 0.6	40.4 ± 0.5
Lignin precipitation mass (g/100 g biomass material)	21.2 ± 0.3	21.5 ± 0.3	16.9 ± 0.3
Lignin separation yield (%)	85.2 ± 1.0	80.2 ± 0.9	81.6 ± 1.0

**Table 3:** Purity analysis of E-DESL, P-DESL, and R-DESL.

Component (%)	E-DESL	P-DESL	R-DESL
Glucose	0.9 ± 0.1	0.7 ± 0.1	0.8 ± 0.1
Xylose	0.4 ± 0.1	0.2 ± 0.1	0.4 ± 0.1
Arabinose	-	-	0.2 ± 0.1
Galactose	-	-	-
Mannose	-	0.2 ± 0.1	-
Acid insoluble lignin (AIL)	90.1 ± 1.0	90.6 ± 1.0	86.9 ± 1.0
Acid soluble lignin (ASL)	1.8 ± 0.1	2.1 ± 0.2	1.6 ± 0.1
Lignin purity *	91.9 ± 1.1	92.7 ± 1.2	88.5 ± 1.1

* Lignin purity is calculated from the sum of AIL (%) and ASL (%) of the DESLs.

**Table 4:** The component dissolved in the mixture during the deep eutectic solvent treatment of biomass feedstocks at 120 °C for 6 h.

Component * (%)	Eucalyptus	Pine	Rice Straw
Glucose	11.1 ± 0.3	9.8 ± 0.3	10.2 ± 0.3
Xylose	77.6 ± 0.6	68.5 ± 0.8	76.5 ± 0.8
Arabinose	73.7 ± 0.7	71.2 ± 0.7	74.8 ± 0.8
Galactose	75.3 ± 0.8	72.6 ± 0.6	73.5 ± 0.8
Mannose	68.2 ± 0.6	78.8 ± 0.8	70.5 ± 0.6

* The percentage of the component dissolved in the mixture to the corresponding component in the biomass feedstocks.

**Table 5:** The elemental analysis of the DESLs.

Lignin Samples	Elemental Analysis (wt %)				C_9_ Formula	H/C_eff_ ^b^	HHV ^c^ (MJ/Kg)
	C	H	O ^a^	N			
E-DESL	61.63	5.45	32.60	0.32	C_9_H_9.6_O_3.6_	0.25	24.43
P-DESL	61.41	5.63	32.84	0.09	C_9_H_9.9_O_3.6_	0.29	24.52
R-DESL	62.33	5.40	31.67	0.55	C_9_H_9.4_O_3.4_	0.25	24.73

^a^ Oxygen content calculated by difference. ^b^ H/C_eff_ = (atomic H-2 × atomic O-3 × atomic N-2)/atomic C. ^c^ The higher heating value (HHV) was estimated: 35.2 C + 116.2 H + 6.3 N +10.5 S − 11.1 O.

**Table 6:** The main oxygen-containing functional group contents of the DESLs.

Functional Groups	Content (mmol/g)		
	E-DESL	P-DESL	R-DESL
Guaiacyl phenolic hydroxyl (G)	1.52	3.09	1.98
Syringyl phenolic hydroxyl (S)	1.71	-	0.62
*p*-hydroxyphenyl phenolic hydroxyl (H)	0.31	0.11	0.99
G/S/H	4.9/5.5/1	-	2.0/0.6/1
Catechol phenolic hydroxyl (C)	0.12	0.26	0.11
Condensed phenolic hydroxyl	1.92	1.78	1.81
Aliphatic hydroxyl (A)	1.93	1.81	1.85
Total phenolic hydroxyl	5.58	5.24	5.51
Total hydroxyl	7.51	7.05	7.36
Carboxyl (-COOH)	1.02	1.05	0.89
Methoxy (-CO_3_)	4.36	3.89	3.67

**Table 7:** Molecular weight analysis of the DESLs.

Lignin Samples	Mn ^a^	Mw ^b^	PDI (Mw/Mn)
E-DESL	1060	1760	1.66
P-DESL	1138	2766	2.43
R-DESL	1056	2280	2.16

^a^ Number of average molecular weight. ^b^ Average molecular weight.

## Data Availability

The data that support the findings of this study are available from the authors upon reasonable request.

## Supplementary Materials

The following supporting information can be downloaded at: https://www.mdpi.com/article/10.3390/molecules29010219/s1. Table S1: The product distributions derived from DESL samples pyrolysis.
