# Supplementary material for: Study on the Relationship between the Structure and Pyrolysis Characteristics of Lignin Isolated from Eucalyptus, Pine, and Rice Straw through the Use of Deep Eutectic Solvent
Source: Molecules. 2023 Dec 30;29(1):219. doi: 10.3390/molecules29010219 (PMC10780212; doi:10.3390/molecules29010219)
Supplement: Supplementary file 1 [file molecules-29-00219-s001.zip › molecules-2770909-supplementary.pdf]

Table S1. The product distributions derived from DESL samples pyrolysis

	Pyrolysis products [a]	CN [b]	Peak area (%)								
			E-DESL			P-DESL			R-DESL		
			400°C	550°C	700°C	400°C	550°C	700°C	400°C	550°C	700°C
G	Guaiacol	C_7	8.82	7.51	6.62	15.01	13.52	9.35	13.52	7.56	5.63
	4-Methylguaiacol	C_8	7.24	11.92	13.37	13.21	14.34	16.32	2.48	7.15	9.86
	Vanillin	C_8	4.52	4.22	3.05	11.64	10.05	6.22	6.56	6.03	4.55
	3-Methylguaiacol	C_8	3.09	2.58	1.56	8.82	7.35	3.28	5.85	5.21	3.57
	4-Vinylguaiacol	C_9	2.89	2.23	2.27	6.79	6.11	4.68	8.94	6.96	4.11
	4-Acetylguaiacol	C_9	3.21	2.52	2.01	5.68	5.43	4.24	4.11	2.96	2.95
	Eugenol	C_{10}	1.21	1.34	1.64	1.94	3.22	3.56	0.73	2.88	4.21
	4-Propylguaiacol	C_{10}	1.36	1.12	0.75	6.85	5.96	1.55	5.22	3.83	2.33
	4-(2-Propanonyl)-guaiacol	C_{10}	5.08	4.81	5.23	9.25	8.52	2.26	7.83	4.33	3.72
	Trans-isoeugenol	C_{10}	0.93	0.64	0.42	3.49	3.04	0.57	2.86	1.86	1.21
	4-Butyrylguaiacol	C_{11}	5.85	5.02	3.22	9.72	8.36	1.77	7.12	4.98	2.79
	**Total**		**44.2**	**44.41**	**40.14**	**92.4**	**85.9**	**53.8**	**65.22**	**53.75**	**44.93**
S	Syringol	C_8	18.52	15.31	12.98	0.7	-	-	3.96	3.69	3.51
	4-Methylsyringol	C_9	13.08	11.82	6.23	-	-	-	3.25	2.54	0.98
	4-Acetylsyringol	C_{10}	5.55	3.03	2.89	-	-	-	2.01	1.31	1.26
	4-Propenylsyringol	C_{11}	7.25	5.83	5.56	-	-	-	2.36	1.31	0.62
	**Total**		**44.4**	**35.99**	**27.66**	**0.7**	**-**	**-**	**11.58**	**8.85**	**6.37**
H	Phenol	C_6	0.72	2.11	3.45	0.70	1.12	4.52	2.16	4.32	7.56
	2-Methylphenol	C_7	0.48	0.96	1.84	-	0.61	2.14	1.21	1.74	2.42
	p-Cresol	C_7	0.16	0.44	0.84	-	0.36	1.64	1.25	2.07	2.57
	2,6-Dimethylphenol	C_8	-	0.08	0.26	-	0.28	1.25	0.55	0.76	0.94
	2-Ethylphenol	C_8	-	-	0.18	-	0.21	1.32	0.89	1.15	1.39
	4-Vinylphenol	C_8	0.12	0.42	0.77	-	0.17	1.28	6.54	10.16	10.44
	2-Ethyl-4-methylphenol	C_9	-	0.16	0.62	-	0.05	0.92	0.69	0.68	0.96
	2-Allylphenol	C_9	0.12	0.33	0.74	-	-	0.87	0.51	0.62	0.61
	2-Methyl-6-(2-propenyl)-phenol	C_{10}	-	-	-	-	-	0.66	-	-	0.39
	2-Allyl-4-methylphenol	C_{10}	-	-	-	-	-	-	-	-	0.32
	**Total**		**1.6**	**4.5**	**8.7**	**0.7**	**2.8**	**14.6**	**13.8**	**21.5**	**27.6**
C	Catechol	C_6	1.41	2.16	5.22	0.82	1.21	6.75	1.35	2.41	3.59
	4-Methylcatechol	C_7	1.26	1.74	3.69	0.48	1.14	4.85	0.96	1.64	2.36
	2,5-dimethylcatechol	C_8	0.74	1.61	2.88	-	0.84	3.64	0.73	1.06	1.58
	4-Ethylcatechol	C_8	0.39	1.19	1.71	-	0.41	2.86	0.56	0.69	1.17
	**Total**		**3.8**	**6.7**	**13.5**	**1.3**	**3.6**	**18.1**	**3.6**	**5.8**	**8.7**

**MAHs**	Toluene	C_7	2.01	2.63	4.51	1.84	3.02	5.61	1.54	2.58	4.55
	o-xylene	C_8	0.52	0.92	1.67	0.86	1.56	2.76	0.69	2.27	2.52
	p-xylene	C_8	0.46	0.63	1.33	0.74	1.28	2.32	0.41	1.86	2.16
	Styrene	C_8	0.31	0.32	0.79	0.36	0.44	1.21	0.26	0.69	0.87
	**Total**		**3.3**	**4.5**	**8.3**	**3.8**	**6.3**	**11.9**	**2.9**	**7.4**	**10.1**
**O**	Acetone	C_3	0.43	0.32	-	0.22	0.06	-	0.48	0.35	0.03
	3-ethyl-2,5-furandione	C_6	0.55	0.51	0.18	0.49	0.38	-	0.55	0.46	0.11
	2-Hydroxy benzaldehyde	C_7	0.31	0.43	0.17	0.12	0.26	0.31	0.31	0.31	0.36
	1-Ethyl-4-methoxy benzene,	C_9	0.42	0.49	0.32	0.16	0.21	0.26	0.44	0.28	0.31
	1-Methoxy-4-(1-methylethyl) benzene	C_{10}	0.46	0.62	0.11	0.08	0.21	0.28	0.38	0.32	0.39
	4-Ethenyl-1,2-dimethoxybenzene	C_{10}	0.31	0.57	0.19	0.03	0.14	0.21	0.26	0.27	0.24
	2-Isopropyl-10-methylphenanthrene	C_{17}	0.22	0.35	0.11	-	0.08	0.24	0.22	0.33	0.41
	1-Methyl-4-p-tolylnaphthalene	C_{18}	-	0.32	0.34	-	-	0.18	0.26	0.38	0.34
	4,4'-Diisopropylbiphenyl	C_{18}	-	0.29	0.28	-	0.06	0.12	-	-	0.11
	**Total**		**2.7**	**3.9**	**1.7**	**1.1**	**1.4**	**1.6**	**2.9**	**2.7**	**2.3**

[a] G: guaiacyl type phenols; S: syringyl type phenols; H: p-hydroxyphenyl type phenols; C: catechol type phenols; M (MAHs): monomeric aromatic hydrocarbons; O: other pyrolysis products

[b] CN: carbon number
